# 4-[(Anthracen-9-yl­methyl­idene)amino]-1,5-dimethyl-2-phenyl-1*H*-pyrazol-3(2*H*)-one

**DOI:** 10.1107/S1600536811029126

**Published:** 2011-07-30

**Authors:** Abdullah M. Asiri, Salman A. Khan, M. Nawaz Tahir

**Affiliations:** a Department of Chemistry, Faculty of Science, King Abduaziz University, Jeddah 21589, PO Box 80203, Saudi Arabia; b University of Sargodha, Department of Physics, Sargodha, Pakistan

## Abstract

In the title compound, C_26_H_21_N_3_O, the phenyl ring of the 4-amino­anti­pyrine group and the heterocyclic five-membered ring along with its substituents, except for the N-bound methyl group (r.m.s. deviation = 0.0027 Å), form a dihedral angle of 54.20 (5)°. Two *S*(6) ring motifs are formed due to intra­molecular C—H⋯N and C—H⋯O hydrogen bonds. In the crystal, mol­ecules are linked into supra­molecular chains along the *a*-axis direction *via* C—H⋯O contacts.

## Related literature

For background to pyrazol-3-ones, see: Asiri & Khan (2010 ▶); Crane *et al.* (1985 ▶); Desai *et al.* (2010 ▶); Rai *et al.* (2009 ▶); Takagi *et al.* (1987 ▶); Yao *et al.* (2007 ▶); Zhang *et al.* (2005 ▶); For related crystal structures, see: Li & Zhang (2006 ▶). For graph-set notation, see: Bernstein *et al.* (1995 ▶).
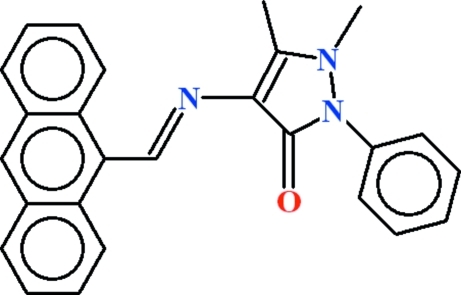


## Experimental

### 

#### Crystal data

C_26_H_21_N_3_O
*M* *_r_* = 391.46
Monoclinic, 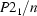

*a* = 7.6603 (3) Å
*b* = 16.4549 (6) Å
*c* = 15.8849 (6) Å
β = 95.243 (1)°
*V* = 1993.91 (13) Å^3^
*Z* = 4
Mo *K*α radiation
μ = 0.08 mm^−1^
*T* = 296 K
0.32 × 0.24 × 0.22 mm

#### Data collection

Bruker Kappa APEXII CCD diffractometer
Absorption correction: multi-scan (*SADABS*; Bruker, 2005 ▶) *T*_min_ = 0.975, *T*_max_ = 0.980
14673 measured reflections
3593 independent reflections
2791 reflections with *I* > 2σ(*I*)
*R*_int_ = 0.039

#### Refinement

*R*[*F* ^2^ > 2σ(*F* ^2^)] = 0.039
*wR*(*F* ^2^) = 0.109
*S* = 1.06
3593 reflections
273 parameters
H-atom parameters constrained
Δρ_max_ = 0.15 e Å^−3^
Δρ_min_ = −0.23 e Å^−3^

### 

Data collection: *APEX2* (Bruker, 2009 ▶); cell refinement: *SAINT* (Bruker, 2009 ▶); data reduction: *SAINT*; program(s) used to solve structure: *SHELXS97* (Sheldrick, 2008 ▶); program(s) used to refine structure: *SHELXL97* (Sheldrick, 2008 ▶); molecular graphics: *ORTEP-3 for Windows* (Farrugia, 1997 ▶) and *PLATON* (Spek, 2009 ▶); software used to prepare material for publication: *WinGX* (Farrugia, 1999 ▶) and *PLATON*.

## Supplementary Material

Crystal structure: contains datablock(s) global, I. DOI: 10.1107/S1600536811029126/tk2768sup1.cif

Structure factors: contains datablock(s) I. DOI: 10.1107/S1600536811029126/tk2768Isup2.hkl

Supplementary material file. DOI: 10.1107/S1600536811029126/tk2768Isup3.cml

Additional supplementary materials:  crystallographic information; 3D view; checkCIF report

## Figures and Tables

**Table 1:** Hydrogen-bond geometry (Å, °)

*D*—H⋯*A*	*D*—H	H⋯*A*	*D*⋯*A*	*D*—H⋯*A*
C5—H5a⋯O1^i^	0.96	2.59	3.530 (2)	167
C5—H5c⋯O1^ii^	0.96	2.57	3.5305 (19)	177
C12—H12⋯O1	0.93	2.37	3.0375 (19)	128
C15—H15⋯N1	0.93	2.42	3.024 (2)	123

Symmetry codes: (i) 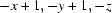; (ii) 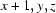.

**Fig. 1.**
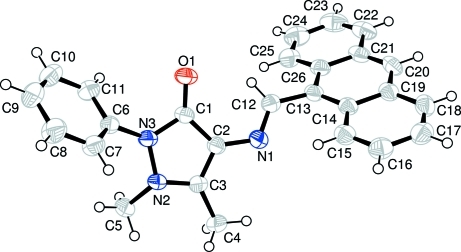
View of the title compound with the atom numbering scheme. The anisotropic displacement ellipsoids are drawn at the 50% probability level. H-atoms are shown as small spheres of arbitrary radii.

**Fig. 2.**
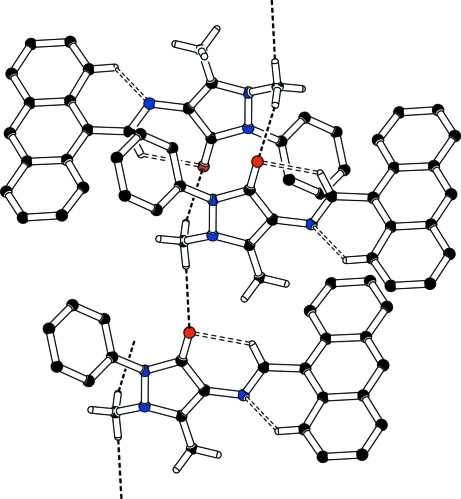
Partial packing diagram showing connectivity between molecules (dashed lines). H atoms not involved in intermolecular interactions are omitted for clarity.

## supplementary crystallographic information

### Comment

Pyrazol-3-one rings are key substructures in a large variety of compounds of therapeutic importance (Zhang *et al.*, 2005). Compounds containing this ring system are known to display diverse pharmacological activities such as analgesic (Takagi *et al.*, 1987), antidepressant (Yao *et al.*, 2007), antibacterial (Rai *et al.*, 2009), plant growth regulatory (Crane *et al.*, 1985) and anti-inflammatory (Desai *et al.*, 2010). Pyrazol-3-one containing Schiff base derivatives dramatically increase the biological activity of the original pyrazol-3-one (Asiri & Khan, 2010). The title compound (I, Fig. 1) has been prepared as a pharmaceutical intermediate. The crystal structures of 1,5-dimethy-4-((2-nitrobenzylidene)amino)-2-phenyl- 1*H*-pyrazol-3(4*H*)-one which is related to (I) has been published (Li & Zhang, 2006).

In (I), the group A (C1/C2/C3/C4/N1/N2/N3/O1) and the benzene ring B (C6—C11) of 4-aminoantipyrine moiety are planar with a r.m.s. deviations of 0.0577 and 0.0027 Å, respectively. The group C (C12—C26) of anthracene-9-carbaldehyde moiety is also planar with an r.m.s. deviation of 0.0665 Å. The dihedral angles between A/B, A/C and B/C are 54.20 (5), 64.07 (2) and 44.43 (5) °, respectively. The methyl group attached at the N atom lies at a distance of 0.5611 (21) Å from the mean plane of A. Intramolecular H-bonding of the type C—H···N and C—H···O complete S(6) ring (Fig. 2 & Table 1) motifs (Bernstein *et al.*, 1995). Intermolecular H-bonding of the type C—H···O sees methyl-H bridging the O atoms to connect molecules into a surpramolecular chain along the *a* axis (Table 1).

### Experimental

A mixture of anthracene-9-carbaldehyde (0.50 g, 2.40 mmol) and 4-aminoantipyrine (0.49 g, 2.40 mmol) in ethanol (15 ml) was heated for 3 h. The progress of the reaction was monitored by TLC. The solid that separated from the cooled mixture was collected and recrystallized from a methanol-chloroform mixture (8:2) to give the yellow prisms of the title compound (I). Yield: 87%; *M*.pt. 404–405 K. IR (KBr) ν_max_ cm^-1^: 3027 (Ar—H), 2874 (C—H), 1636 (C═O), 1580 (HC═N), 1138 (C—N). ^1^H NMR (600 MHz, CDCl_3_) (δ p.p.m.): 11.06 (s, CH_olefinic_), 8.98–7.36 (m,14H, Ar—H) 3.23 (s, CH_3_), 2.19 (s, CH_3_).

### Refinement

The H-atoms were positioned geometrically (C–H = 0.93–0.96 Å) and refined as riding with *U*_iso_(H) = x*U*_eq_(C), where x = 1.5 for methyl and x = 1.2 for aryl H-atoms.

### Crystal data

**Table:**

C_26_H_21_N_3_O	*F*(000) = 824
*M**_r_* = 391.46	*D*_x_ = 1.304 Mg m^−^^3^
Monoclinic, *P*2_1_/*n*	Mo *K*α radiation, λ = 0.71073 Å
Hall symbol: -P 2yn	Cell parameters from 2791 reflections
*a* = 7.6603 (3) Å	θ = 2.8–25.3°
*b* = 16.4549 (6) Å	µ = 0.08 mm^−^^1^
*c* = 15.8849 (6) Å	*T* = 296 K
β = 95.243 (1)°	Prism, yellow
*V* = 1993.91 (13) Å^3^	0.32 × 0.24 × 0.22 mm
*Z* = 4	

### Data collection

**Table:**

Bruker Kappa APEXII CCD diffractometer	3593 independent reflections
Radiation source: fine-focus sealed tube	2791 reflections with *I* > 2σ(*I*)
graphite	*R*_int_ = 0.039
Detector resolution: 8.10 pixels mm^-1^	θ_max_ = 25.3°, θ_min_ = 2.8°
ω scans	*h* = −9→9
Absorption correction: multi-scan (*SADABS*; Bruker, 2005)	*k* = −19→19
*T*_min_ = 0.975, *T*_max_ = 0.980	*l* = −19→19
14673 measured reflections	

### Refinement

**Table:**

Refinement on *F*^2^	Primary atom site location: structure-invariant direct methods
Least-squares matrix: full	Secondary atom site location: difference Fourier map
*R*[*F*^2^ > 2σ(*F*^2^)] = 0.039	Hydrogen site location: inferred from neighbouring sites
*wR*(*F*^2^) = 0.109	H-atom parameters constrained
*S* = 1.06	*w* = 1/[σ^2^(*F*_o_^2^) + (0.0463*P*)^2^ + 0.4551*P*] where *P* = (*F*_o_^2^ + 2*F*_c_^2^)/3
3593 reflections	(Δ/σ)_max_ < 0.001
273 parameters	Δρ_max_ = 0.15 e Å^−^^3^
0 restraints	Δρ_min_ = −0.23 e Å^−^^3^

### Special details

**Table:**

Geometry. Bond distances, angles *etc*. have been calculated using the rounded fractional coordinates. All su's are estimated from the variances of the (full) variance-covariance matrix. The cell e.s.d.'s are taken into account in the estimation of distances, angles and torsion angles
Refinement. Refinement of *F*^2^ against ALL reflections. The weighted *R*-factor *wR* and goodness of fit *S* are based on *F*^2^, conventional *R*-factors *R* are based on *F*, with *F* set to zero for negative *F*^2^. The threshold expression of *F*^2^ > σ(*F*^2^) is used only for calculating *R*-factors(gt) *etc*. and is not relevant to the choice of reflections for refinement. *R*-factors based on *F*^2^ are statistically about twice as large as those based on *F*, and *R*- factors based on ALL data will be even larger.

### Fractional atomic coordinates and isotropic or equivalent isotropic displacement parameters (Å^2^)

**Table:**

	*x*	*y*	*z*	*U*_iso_*/*U*_eq_	
O1	0.30051 (14)	0.39403 (7)	0.06517 (7)	0.0520 (4)	
N1	0.56061 (17)	0.42547 (8)	0.22734 (8)	0.0409 (4)	
N2	0.74133 (15)	0.37942 (7)	0.03382 (7)	0.0359 (4)	
N3	0.56279 (15)	0.38171 (8)	0.00463 (8)	0.0383 (4)	
C1	0.46213 (19)	0.39626 (9)	0.07155 (9)	0.0367 (5)	
C2	0.58906 (19)	0.41001 (9)	0.14336 (9)	0.0359 (5)	
C3	0.75240 (19)	0.40245 (8)	0.11629 (9)	0.0348 (5)	
C4	0.9234 (2)	0.42095 (10)	0.16435 (10)	0.0465 (6)	
C5	0.8631 (2)	0.40778 (10)	−0.02533 (10)	0.0441 (5)	
C6	0.50243 (18)	0.34201 (9)	−0.07195 (9)	0.0352 (5)	
C7	0.5734 (2)	0.26909 (10)	−0.09414 (10)	0.0460 (5)	
C8	0.5114 (3)	0.23253 (12)	−0.16899 (12)	0.0600 (7)	
C9	0.3794 (3)	0.26782 (13)	−0.22057 (12)	0.0624 (7)	
C10	0.3093 (3)	0.34023 (12)	−0.19815 (11)	0.0574 (6)	
C11	0.3707 (2)	0.37817 (10)	−0.12373 (10)	0.0457 (5)	
C12	0.4106 (2)	0.44921 (10)	0.24467 (10)	0.0442 (5)	
C13	0.36155 (19)	0.46454 (9)	0.33093 (9)	0.0396 (5)	
C14	0.40579 (19)	0.41027 (9)	0.39838 (9)	0.0391 (5)	
C15	0.5113 (2)	0.33949 (10)	0.39171 (11)	0.0461 (6)	
C16	0.5522 (2)	0.29004 (11)	0.45886 (11)	0.0527 (6)	
C17	0.4888 (2)	0.30582 (12)	0.53761 (11)	0.0551 (7)	
C18	0.3861 (2)	0.37129 (11)	0.54708 (10)	0.0511 (6)	
C19	0.3405 (2)	0.42567 (10)	0.47887 (10)	0.0427 (5)	
C20	0.2374 (2)	0.49383 (10)	0.48880 (11)	0.0477 (5)	
C21	0.1963 (2)	0.54873 (10)	0.42364 (11)	0.0455 (5)	
C22	0.0966 (2)	0.62024 (11)	0.43539 (13)	0.0570 (7)	
C23	0.0667 (3)	0.67545 (12)	0.37312 (15)	0.0661 (7)	
C24	0.1354 (3)	0.66362 (12)	0.29507 (14)	0.0648 (7)	
C25	0.2282 (2)	0.59577 (11)	0.28014 (12)	0.0542 (6)	
C26	0.2605 (2)	0.53462 (10)	0.34333 (10)	0.0423 (5)	
H4A	1.00701	0.37971	0.15337	0.0698*	
H4B	0.90884	0.42224	0.22373	0.0698*	
H4C	0.96475	0.47284	0.14700	0.0698*	
H5A	0.83578	0.46297	−0.04108	0.0661*	
H5B	0.85281	0.37406	−0.07485	0.0661*	
H5C	0.98079	0.40495	0.00106	0.0661*	
H7	0.66211	0.24477	−0.05898	0.0552*	
H8	0.55952	0.18352	−0.18466	0.0720*	
H9	0.33756	0.24253	−0.27077	0.0749*	
H10	0.21973	0.36406	−0.23322	0.0689*	
H11	0.32363	0.42763	−0.10874	0.0548*	
H12	0.32552	0.45754	0.19988	0.0530*	
H15	0.55291	0.32700	0.34006	0.0554*	
H16	0.62332	0.24500	0.45278	0.0632*	
H17	0.51757	0.27122	0.58306	0.0661*	
H18	0.34431	0.38108	0.59928	0.0614*	
H20	0.19451	0.50300	0.54088	0.0572*	
H22	0.05130	0.62897	0.48699	0.0683*	
H23	0.00031	0.72151	0.38185	0.0793*	
H24	0.11711	0.70285	0.25306	0.0778*	
H25	0.27146	0.58896	0.22778	0.0650*	

### Atomic displacement parameters (Å^2^)

**Table:**

	*U*^11^	*U*^22^	*U*^33^	*U*^12^	*U*^13^	*U*^23^
O1	0.0282 (6)	0.0734 (8)	0.0544 (7)	0.0007 (5)	0.0042 (5)	−0.0123 (6)
N1	0.0352 (7)	0.0483 (8)	0.0397 (7)	−0.0023 (6)	0.0060 (5)	−0.0023 (6)
N2	0.0254 (6)	0.0435 (7)	0.0390 (7)	−0.0014 (5)	0.0037 (5)	−0.0011 (5)
N3	0.0274 (7)	0.0475 (8)	0.0399 (7)	−0.0002 (5)	0.0020 (5)	−0.0062 (5)
C1	0.0292 (8)	0.0389 (9)	0.0425 (8)	−0.0003 (6)	0.0054 (6)	−0.0030 (6)
C2	0.0323 (8)	0.0381 (8)	0.0376 (8)	−0.0003 (6)	0.0050 (6)	−0.0008 (6)
C3	0.0317 (8)	0.0339 (8)	0.0386 (8)	−0.0001 (6)	0.0024 (6)	0.0032 (6)
C4	0.0322 (9)	0.0613 (11)	0.0454 (9)	−0.0030 (7)	−0.0004 (7)	0.0024 (8)
C5	0.0355 (9)	0.0536 (10)	0.0442 (9)	−0.0064 (7)	0.0099 (7)	0.0016 (7)
C6	0.0308 (8)	0.0383 (8)	0.0368 (8)	−0.0049 (6)	0.0042 (6)	0.0000 (6)
C7	0.0417 (9)	0.0468 (9)	0.0483 (9)	0.0043 (7)	−0.0025 (7)	−0.0027 (7)
C8	0.0597 (12)	0.0573 (11)	0.0612 (11)	0.0043 (9)	−0.0037 (9)	−0.0193 (9)
C9	0.0607 (12)	0.0750 (14)	0.0493 (11)	−0.0072 (10)	−0.0068 (9)	−0.0173 (9)
C10	0.0524 (11)	0.0669 (12)	0.0500 (10)	0.0003 (9)	−0.0115 (8)	0.0050 (9)
C11	0.0429 (9)	0.0444 (9)	0.0488 (10)	0.0022 (7)	−0.0009 (7)	0.0019 (7)
C12	0.0365 (9)	0.0544 (10)	0.0413 (9)	0.0018 (7)	0.0014 (7)	−0.0063 (7)
C13	0.0305 (8)	0.0477 (9)	0.0410 (8)	−0.0048 (7)	0.0049 (6)	−0.0077 (7)
C14	0.0310 (8)	0.0445 (9)	0.0420 (8)	−0.0087 (7)	0.0043 (6)	−0.0078 (7)
C15	0.0422 (10)	0.0484 (10)	0.0481 (9)	−0.0030 (7)	0.0056 (7)	−0.0069 (7)
C16	0.0457 (10)	0.0494 (10)	0.0623 (11)	−0.0039 (8)	0.0010 (8)	0.0002 (8)
C17	0.0486 (11)	0.0597 (12)	0.0555 (11)	−0.0140 (9)	−0.0029 (8)	0.0105 (9)
C18	0.0471 (10)	0.0657 (12)	0.0410 (9)	−0.0189 (9)	0.0064 (7)	−0.0024 (8)
C19	0.0352 (9)	0.0499 (10)	0.0436 (9)	−0.0138 (7)	0.0063 (7)	−0.0070 (7)
C20	0.0398 (9)	0.0580 (10)	0.0477 (9)	−0.0128 (8)	0.0173 (7)	−0.0150 (8)
C21	0.0317 (8)	0.0487 (10)	0.0572 (10)	−0.0086 (7)	0.0109 (7)	−0.0131 (8)
C22	0.0396 (10)	0.0558 (11)	0.0774 (13)	−0.0030 (8)	0.0159 (9)	−0.0201 (10)
C23	0.0450 (11)	0.0522 (11)	0.1008 (16)	0.0055 (9)	0.0058 (10)	−0.0150 (11)
C24	0.0511 (12)	0.0590 (12)	0.0829 (14)	0.0060 (9)	−0.0016 (10)	0.0047 (10)
C25	0.0443 (10)	0.0623 (11)	0.0554 (10)	0.0036 (8)	0.0015 (8)	−0.0019 (9)
C26	0.0295 (8)	0.0487 (9)	0.0489 (9)	−0.0042 (7)	0.0041 (7)	−0.0081 (7)

### Geometric parameters (Å, °)

**Table:**

O1—C1	1.2335 (18)	C21—C22	1.424 (2)
N1—C2	1.3946 (19)	C21—C26	1.428 (2)
N1—C12	1.268 (2)	C22—C23	1.347 (3)
N2—N3	1.4039 (16)	C23—C24	1.404 (3)
N2—C3	1.3589 (18)	C24—C25	1.356 (3)
N2—C5	1.4598 (19)	C25—C26	1.427 (2)
N3—C1	1.3899 (19)	C4—H4A	0.9600
N3—C6	1.4204 (19)	C4—H4B	0.9600
C1—C2	1.447 (2)	C4—H4C	0.9600
C2—C3	1.365 (2)	C5—H5A	0.9600
C3—C4	1.486 (2)	C5—H5B	0.9600
C6—C7	1.376 (2)	C5—H5C	0.9600
C6—C11	1.377 (2)	C7—H7	0.9300
C7—C8	1.378 (3)	C8—H8	0.9300
C8—C9	1.371 (3)	C9—H9	0.9300
C9—C10	1.367 (3)	C10—H10	0.9300
C10—C11	1.381 (2)	C11—H11	0.9300
C12—C13	1.475 (2)	C12—H12	0.9300
C13—C14	1.412 (2)	C15—H15	0.9300
C13—C26	1.413 (2)	C16—H16	0.9300
C14—C15	1.427 (2)	C17—H17	0.9300
C14—C19	1.437 (2)	C18—H18	0.9300
C15—C16	1.355 (2)	C20—H20	0.9300
C16—C17	1.407 (2)	C22—H22	0.9300
C17—C18	1.351 (3)	C23—H23	0.9300
C18—C19	1.424 (2)	C24—H24	0.9300
C19—C20	1.389 (2)	C25—H25	0.9300
C20—C21	1.388 (2)		
			
C2—N1—C12	119.21 (13)	C13—C26—C21	119.69 (14)
N3—N2—C3	106.50 (11)	C13—C26—C25	122.66 (14)
N3—N2—C5	116.06 (11)	C21—C26—C25	117.53 (15)
C3—N2—C5	122.96 (12)	C3—C4—H4A	109.00
N2—N3—C1	110.11 (11)	C3—C4—H4B	109.00
N2—N3—C6	120.10 (11)	C3—C4—H4C	109.00
C1—N3—C6	125.05 (12)	H4A—C4—H4B	109.00
O1—C1—N3	123.70 (13)	H4A—C4—H4C	109.00
O1—C1—C2	131.78 (14)	H4B—C4—H4C	109.00
N3—C1—C2	104.48 (12)	N2—C5—H5A	109.00
N1—C2—C1	129.04 (13)	N2—C5—H5B	109.00
N1—C2—C3	123.07 (13)	N2—C5—H5C	109.00
C1—C2—C3	107.85 (13)	H5A—C5—H5B	109.00
N2—C3—C2	110.53 (13)	H5A—C5—H5C	109.00
N2—C3—C4	121.79 (13)	H5B—C5—H5C	109.00
C2—C3—C4	127.56 (13)	C6—C7—H7	120.00
N3—C6—C7	121.00 (13)	C8—C7—H7	120.00
N3—C6—C11	118.43 (13)	C7—C8—H8	120.00
C7—C6—C11	120.57 (14)	C9—C8—H8	120.00
C6—C7—C8	119.25 (15)	C8—C9—H9	120.00
C7—C8—C9	120.54 (18)	C10—C9—H9	120.00
C8—C9—C10	119.95 (18)	C9—C10—H10	120.00
C9—C10—C11	120.38 (18)	C11—C10—H10	120.00
C6—C11—C10	119.31 (16)	C6—C11—H11	120.00
N1—C12—C13	124.56 (14)	C10—C11—H11	120.00
C12—C13—C14	122.19 (13)	N1—C12—H12	118.00
C12—C13—C26	117.28 (13)	C13—C12—H12	118.00
C14—C13—C26	120.47 (13)	C14—C15—H15	119.00
C13—C14—C15	123.92 (14)	C16—C15—H15	119.00
C13—C14—C19	118.98 (13)	C15—C16—H16	119.00
C15—C14—C19	117.09 (14)	C17—C16—H16	120.00
C14—C15—C16	121.55 (15)	C16—C17—H17	120.00
C15—C16—C17	121.01 (16)	C18—C17—H17	120.00
C16—C17—C18	119.88 (16)	C17—C18—H18	119.00
C17—C18—C19	121.43 (15)	C19—C18—H18	119.00
C14—C19—C18	119.01 (14)	C19—C20—H20	119.00
C14—C19—C20	119.41 (15)	C21—C20—H20	119.00
C18—C19—C20	121.57 (15)	C21—C22—H22	119.00
C19—C20—C21	122.23 (15)	C23—C22—H22	119.00
C20—C21—C22	121.85 (16)	C22—C23—H23	120.00
C20—C21—C26	119.16 (15)	C24—C23—H23	120.00
C22—C21—C26	118.95 (16)	C23—C24—H24	120.00
C21—C22—C23	121.21 (18)	C25—C24—H24	120.00
C22—C23—C24	120.17 (19)	C24—C25—H25	119.00
C23—C24—C25	120.89 (19)	C26—C25—H25	119.00
C24—C25—C26	121.17 (17)		
			
C12—N1—C2—C1	−17.9 (2)	N1—C12—C13—C26	137.66 (17)
C12—N1—C2—C3	164.62 (15)	C12—C13—C14—C15	4.7 (2)
C2—N1—C12—C13	177.51 (14)	C12—C13—C14—C19	−174.64 (14)
C3—N2—N3—C1	−7.39 (15)	C26—C13—C14—C15	−178.17 (15)
C3—N2—N3—C6	−164.20 (12)	C26—C13—C14—C19	2.5 (2)
C5—N2—N3—C1	−148.34 (13)	C12—C13—C26—C21	174.53 (14)
C5—N2—N3—C6	54.85 (17)	C12—C13—C26—C25	−9.7 (2)
N3—N2—C3—C2	7.04 (15)	C14—C13—C26—C21	−2.8 (2)
N3—N2—C3—C4	−169.32 (13)	C14—C13—C26—C25	173.05 (15)
C5—N2—C3—C2	144.62 (13)	C13—C14—C15—C16	178.95 (15)
C5—N2—C3—C4	−31.7 (2)	C19—C14—C15—C16	−1.7 (2)
N2—N3—C1—O1	−173.21 (14)	C13—C14—C19—C18	−179.55 (14)
N2—N3—C1—C2	4.81 (16)	C13—C14—C19—C20	−0.7 (2)
C6—N3—C1—O1	−17.8 (2)	C15—C14—C19—C18	1.1 (2)
C6—N3—C1—C2	160.23 (13)	C15—C14—C19—C20	179.89 (15)
N2—N3—C6—C7	36.9 (2)	C14—C15—C16—C17	1.3 (2)
N2—N3—C6—C11	−143.11 (14)	C15—C16—C17—C18	−0.3 (3)
C1—N3—C6—C7	−116.22 (17)	C16—C17—C18—C19	−0.4 (3)
C1—N3—C6—C11	63.7 (2)	C17—C18—C19—C14	−0.1 (2)
O1—C1—C2—N1	−0.5 (3)	C17—C18—C19—C20	−178.87 (16)
O1—C1—C2—C3	177.30 (16)	C14—C19—C20—C21	−0.8 (2)
N3—C1—C2—N1	−178.28 (15)	C18—C19—C20—C21	177.99 (15)
N3—C1—C2—C3	−0.50 (16)	C19—C20—C21—C22	−177.33 (15)
N1—C2—C3—N2	173.80 (13)	C19—C20—C21—C26	0.6 (2)
N1—C2—C3—C4	−10.1 (2)	C20—C21—C22—C23	175.93 (18)
C1—C2—C3—N2	−4.15 (16)	C26—C21—C22—C23	−2.0 (3)
C1—C2—C3—C4	171.95 (14)	C20—C21—C26—C13	1.2 (2)
N3—C6—C7—C8	−179.94 (16)	C20—C21—C26—C25	−174.80 (15)
C11—C6—C7—C8	0.1 (2)	C22—C21—C26—C13	179.17 (15)
N3—C6—C11—C10	−179.52 (15)	C22—C21—C26—C25	3.2 (2)
C7—C6—C11—C10	0.4 (2)	C21—C22—C23—C24	−0.6 (3)
C6—C7—C8—C9	−0.6 (3)	C22—C23—C24—C25	1.9 (3)
C7—C8—C9—C10	0.6 (3)	C23—C24—C25—C26	−0.6 (3)
C8—C9—C10—C11	0.0 (3)	C24—C25—C26—C13	−177.82 (17)
C9—C10—C11—C6	−0.5 (3)	C24—C25—C26—C21	−1.9 (2)
N1—C12—C13—C14	−45.1 (2)		

### Hydrogen-bond geometry (Å, °)

**Table:**

*D*—H···*A*	*D*—H	H···*A*	*D*···*A*	*D*—H···*A*
C5—H5a···O1^i^	0.96	2.59	3.530 (2)	167
C5—H5c···O1^ii^	0.96	2.57	3.5305 (19)	177
C12—H12···O1	0.93	2.37	3.0375 (19)	128
C15—H15···N1	0.93	2.42	3.024 (2)	123

Symmetry codes: (i) −*x*+1, −*y*+1, −*z*; (ii) *x*+1, *y*, *z*.
